# Frequent Disengagement and Subsequent Mortality Among People With HIV and Hepatitis C in Canada: A Prospective Cohort Study

**DOI:** 10.1093/ofid/ofae239

**Published:** 2024-04-25

**Authors:** Sahar Saeed, Tyler Thomas, Duy A Dinh, Erica Moodie, Joseph Cox, Curtis Cooper, John Gill, Valerie Martel-Laferriere, Dimitra Panagiotoglou, Sharon Walmsley, Alexander Wong, Marina B Klein

**Affiliations:** Department of Public Health Sciences, Queen's University, Kingston, Ontario, Canada; Department of Public Health Sciences, Queen's University, Kingston, Ontario, Canada; Department of Public Health Sciences, Queen's University, Kingston, Ontario, Canada; School of Population and Global Health, McGill University, Montreal, Quebec, Canada; School of Population and Global Health, McGill University, Montreal, Quebec, Canada; Department of Medicine, University of Ottawa, Ottawa, Ontario, Canada; Department of Medicine, University of Calgary, Calgary, Alberta, Canada; Department of Medicine, Centre de recherche du Centre hospitalier de l'Université Montréal, Montreal, Quebec, Canada; Department of Medicine, Centre de recherche du Centre hospitalier de l'Université Montréal, Montreal, Quebec, Canada; School of Population and Global Health, McGill University, Montreal, Quebec, Canada; Department of Medicine, University Health Network, Toronto, Ontario, Canada; Department of Medicine, Regina Qu’Appelle Health Region, Regina, Saskatchewan, Canada; Department of Medicine, Division of Infectious Diseases/Chronic Viral Illness Service, McGill University Health Center, Quebec, Canada; Canadian HIV Trials Network, Vancouver, British Columbia, Canada

**Keywords:** cascade-of-care, disengagement, HIV-Hepatitis C co-infection, mortality, lost-to-follow-up

## Abstract

**Background:**

The cascade of care, commonly used to assess HIV and hepatitis C (HCV) health service delivery, has limitations in capturing the complexity of individuals’ engagement patterns. This study examines the dynamic nature of engagement and mortality trajectories among people with HIV and HCV.

**Methods:**

We used data from the Canadian HIV-HCV Co-Infection Cohort, which prospectively follows 2098 participants from 18 centers biannually. Markov multistate models were used to evaluate sociodemographic and clinical factors associated with transitioning between the following states: (1) lost-to-follow-up (LTFU), defined as no visit for 18 months; (2) reengaged (reentry into cohort after being LTFU); (3) withdrawn from the study (ie, moved); (4) death; otherwise remained (5) engaged-in-care.

**Results:**

A total of 1809 participants met the eligibility criteria and contributed 12 591 person-years from 2003 to 2022. LTFU was common, with 46% experiencing at least 1 episode, of whom only 57% reengaged. One in 5 (n = 383) participants died during the study. Participants who transitioned to LTFU were twice as likely to die as those who were consistently engaged. Factors associated with transitioning to LTFU included detectable HCV RNA (adjusted hazards ratio [aHR], 1.37; 95% confidence interval [CI], 1.13–1.67), evidence of HCV treatment but no sustained virologic response result (aHR, 1.99; 95% CI, 1.56–2.53), and recent incarceration (aHR, 1.94; 95% CI, 1.58–2.40). Being Indigenous was a significant predictor of death across all engagement trajectories.

**Interpretation:**

Disengagement from clinical care was common and resulted in higher death rates. People LTFU were more likely to require HCV treatment highlighting a priority population for elimination strategies.

## Teaser

Disengagement from clinical care was common and resulted in higher death rates among people with HIV and hepatitis C. People lost to follow-up were more likely to require hepatitis C virus treatment, highlighting a priority population for elimination strategies.

Hepatitis C (HCV) coinfection is estimated to occur in 20% to 30% of Canadians with HIV [1] and 50% to 90% of people with HIV (PWH) who inject drugs [2]. The social determinants of health associated with injection drug use include a history of trauma, low income/levels of education, transactional sex, food/housing insecurity, and incarceration [3]. In Canada, Indigenous peoples experience a disproportional burden of substance abuse and account for 70% to 80% of new HCV infections among people who inject drugs (PWID) [3]. And in 2020, 25% of all new HIV infections in Canada were among females [4]. The intertwined challenges of HIV, HCV, and injection drug use form a syndemic, exacerbating the clinical outcomes of each condition, widening health disparities and social vulnerabilities [5].

The cascade of care, also known as the care continuum, is used to evaluate the effectiveness of HIV and HCV health service delivery. This point-in-time assessment helps policymakers and health care providers identify areas for improvement by pinpointing gaps in prevention and treatment services. Although conceptually straightforward and helpful when comparing subpopulations or geographical regions, this framework falls short in capturing the complexity and longitudinal nature of individuals’ engagement patterns [6]. This is because the transitions between each step of the care cascade are not always linear; instead, often follow a dynamic and cyclical process [7, 8]. Moreover, “losses” at each step of the cascade include intermittent or permanent reasons (ie, mortality), representing a distinct phenotype of disengagement from clinical care [9]. Other methodological challenges associated with the cascade of care include incomplete data or gaps between visits and inconsistent definitions for metrics such as disengagement [10].

A longitudinal focus on the care continuum may be especially important for PWH and HCV as they follow 2 nested cascades of care. HIV care requires lifetime engagement to maintain sustained HIV viral suppression [11, 12], whereas the HCV cascade of care rarely incorporates steps beyond sustained virologic response (SVR) (or HCV cure), even though reinfection remains a risk [13]. As countries strive to reach HCV elimination targets set by the World Health Organization [14], it is becoming increasingly evident that elimination efforts will be impeded by patients being lost from care before HCV treatment initiation or when SVR is determined [15–17]. And for the populations treated and cured, sustained engagement in care will remain essential to achieve optimal long-term health outcomes [18, 19]. Although many studies have described the cascade of HIV or HCV care, few have taken a comprehensive approach by focusing on retention patterns over time for those living with both viruses. This study aims to contribute to understanding longitudinal care engagement and mortality trajectories among PWH and HCV. Using an advanced analytical approach and individual-level longitudinal data, we explore the dynamic nature of care engagement and evaluate sociodemographic and clinical factors associated with transitioning in and out of clinical care and to death.

## METHODS

### Population and Setting

We analyzed data from the Canadian Co-infection Cohort Study (CCC), a prospective cohort of PWH with evidence of HCV infection recruited from 18 HIV centers across Canada; details on recruitment and procedures have been published elsewhere [20]. Briefly, recruitment centers are in both large urban and smaller cities to include individuals who access various models of care (ie, outreach programs) and have diverse risk profiles (ie, active and former PWID, men who have sex with men [MSM], women, and Indigenous populations) representative of the coinfected population in care in Canada. Since April 2003, participants have consented to complete a detailed questionnaire and laboratory assessments every 6 months. Cohort visits were designed to be paired with routine clinical follow-up, mirroring routine standards of care across Canada. Study coordinators also conduct a standardized chart review to collect data on medical conditions, health care services, and medications. Annually, data are linked to vital registries to obtain information on deaths for all cohort participants known to have died or those lost to follow-up. To date, it is estimated that the CCC has enrolled ∼23% of the total HIV coinfected population in care in Canada.

### Patient Consent Statement

All participants provided written informed consent, and the cohort study (CCC) was approved by all institutional ethics boards of the participating institutions and the community advisory committee of the Canadian HIV Trials Network. This analysis was approved by the research ethics board at Queen's University (REB #6037697).

### Eligibility

CCC participants who completed a baseline visit, followed by at least 1 follow-up visit within 18 months, were considered engaged in care and included in this analysis.

### Outcomes

After meeting the eligibility criteria, participants could remain “engaged-in-care”; defined as maintaining cohort visits within 18 months until administrative censoring (1 October 2022) or participants could transition to 4 mutually exclusive states (1) “lost to follow-up” (LTFU); defined as a gap in visits of greater than 18 months, not as a result of death or withdrawing from the study (2) “reengaged in care”; defined as returning to care after transitioning to at least 1 episode of LTFU returning to care, (3) “withdrawal”; defined as intentionally leaving the cohort for any reason (ie, withdrawing consent to participate in the study or moving, which included transferring medical care to another clinic or moving to another city), or (4) “death.” The reasons for withdrawal were documented. The date and cause of death were confirmed for all CCC participants presumed to have died or were considered lost from clinical care by linking to provincial vial registries and reviewing corners reports. For all identified deaths, standardized case report forms were used to accurately describe the causes of death based on the Coding of Cause of Death in HIV Protocol [21]. Sensitivity analyses were also conducted to evaluate the varying definitions of LTFU at 9-, 12-, or 24-month intervals.

### Covariates

A priori, we chose the following covariates to include in multivariate models. Sociodemographic and key population factors: age (continuous measure by 10-year intervals), self-identification of Indigenous status, biological sex, MSM, income (<$18 000 CAD per year) (time updated), PWID defined as injecting drugs in the last six months (time updated), and incarceration in the past 6 months (time updated). Clinical variables (all time updated) included advanced liver disease (measured as an AST to platelet ratio index score > 1.5), undetectable HIV RNA (<50 copies/mL), CD4 cell count (continuous measure by 100 cells/mm^3^), HCV RNA or missing results, diagnosis of sexually transmitted infection, HCV treatment status categorized as treated and achieved SVR, treated but unknown SVR results, failed treatment or never treated. Calendar time was also included to reflect changes in clinical care and treatments over time.

### Statistical Analysis

Incidence rates between transition states were reported as events per 100 person-years (py) at risk. Markov multistate models were used to model multiple transition states simultaneously [22]. This approach extends beyond traditional survival models as it adequately accounts for the competing risks to other states. The data set was formulated to consider all possible transitions a participant was at risk for any of the 5 outcomes. By censoring participants for all possible transitions that were not observed at the event time of the observed transition, we accounted for competing risks. Information loss is minimized by choosing not to discretize time, and transitions are recorded at observed event times. Multistate models also sufficiently accommodate censoring and time-varying covariates. Models assume a form identical to Cox proportional hazards models, with a baseline intensity function and multiplicative covariate effects. Exponentiated regression coefficients yield intensity ratio or hazard ratio interpretations. Missing data were imputed using multiple imputations by chained equations by classification regression trees (to account for categorical/continuous variables). Nonlinear trends in transition intensity were identified over the follow-up time. Calendar year was modeled as a nonlinear function by a penalized smoothing spline with evenly spaced basis functions (Supplementary Figure 1). A frailty term (random effect) was included in our model to account for the variability in clinical care for each center (n = 18). All analysis was conducted using R.

## RESULTS

Of 2098 CCC participants enrolled between April 2003 and October 2022, 1809 met our eligibility criteria and contributed 12 591 py. At baseline (Table 1), the median age was aged 45 years, 419 (23%) identified as MSM, 1483 (82%) reported past injection drug use, 542 (30%) were active PWID, 449 (25%) as Indigenous, and 514 (28%) as women. Clinically, 1167 (65%) of the cohort had a suppressed HIV viral load with a median CD4 cell count of 418 cells/mm^3^; 316 (17%) reported past HCV treatment; 203 (11%) had advanced liver fibrosis, and 91 (5%) had a recent diagnosis of a sexually transmitted infection.

**Table 1.** Baseline Characteristics of the CCC Participants Between 2003 and 2022

		Total = 1809 N (%)
Sex	Male	1275 (70.5)
	Female	514 (28.4)
	Trans	13 (0.7)
	Missing	7 (0.4)
Age, y	Mean (SD)	44.8 (9.4)
	Missing	1 (0.1)
Province	British Columbia	545 (30.1)
	Alberta	44 (2.4)
	Saskatchewan	215 (11.9)
	Ontario	419 (23.2)
	Quebec	586 (32.4)
Indigenous	Yes	449 (24.8)
	Missing	94 (5.2)
Men who have sex with men	Yes	419 (23.2)
	Missing	15 (0.8)
People who inject drugs in the past 6 mo	Yes	542 (30)
	Missing	8 (0.4)
Low income (<$1500/mo)	Yes	1368 (75.6)
	Missing	26 (1.4)
Incarceration (in the past 6 mo)	Yes	210 (11.5)
	Missing	195 (10.8)
HIV viral load undetectable (<50 copies)	Yes	1167 (65)
	Missing	90 (5)
CD4 cell count (cells/mm^3^)	Mean (SD)	457 (279)
	Missing	36 (2.0)
STI (diagnosis in past 6 mo)	Yes	91 (5)
	Missing	138 (7.6)
Advanced fibrosis (APRI >1.5)	Yes	203 (11.2)
	Missing	531 (29.4)
HCV RNA (detectable)	Yes	1194 (66)
	Missing/not done	441 (24.4)
HCV treatment	Sustained virologic response	120 (6.6)
	Failed treatment	45 (2.5)
	Missing treatment results	151 (8.3)
	Not treated	1493 (82.5)
Year of entry into cohort	2003–2007	127 (7)
	2007–2014	731 (40.4)
	2014–2020	863 (47.7)
	2020–2022	88 (4.9)

Abbreviations: APRI, AST to platelet ratio index; CCC, Canadian Co-infection Cohort Study; HCV, hepatitis C virus; SD, standard deviation; STI, sexually transmitted infection.

**Figure 1.**
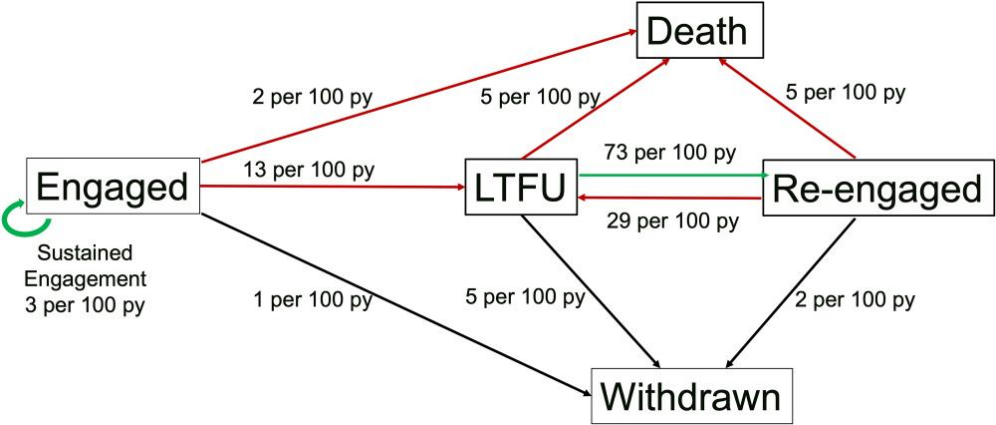
Incidence rates (events per 100 person-years [py]. Red arrows represent negative transitions to death or lost-to-follow-up [LTFU]), positive transitions include remaining engaged or re-engaging in care (green arrows). Engaged is defined as maintaining cohort visits within 18 months until administrative censoring. LTFU is defined as a gap in visits of longer than 18 months, not because of death or withdrawal from the study. Reengaged is defined as returning to care after transitioning to at least 1 episode of LTFU returning to care. Withdrawn is defined as intentionally leaving the cohort for any reason (ie, withdrawing consent to participate in the study, changing providers or moving to another city).

LTFU was common in the study population; 831 (46%) participants had 1 LTFU episode, 296 (16%) had 2 episodes, 85 (5%) had 3 episodes, 13 (<1%) had 4, and 1 had 5 episodes of being LTFU. Of the 1226 LTFU events, 697 (57%) reengaged. Figure 1 illustrates incidence rates of transitioning between five transition states per 100 py at risk; similar trends in the results were observed using alternative definitions of LTFU (Supplementary Table 1). There was significant movement in and out of care; 13 events per 100 py transitioning from engaged to LTFU, 73 events per 100 py from LTFU to reengaged, and 29 per 100 py from reengaged to LTFU. Participants were more likely to withdraw from the cohort from the LTFU state (5 per 100 py) compared to those engaged (1 per 100 py) or reengaged (2 per 100 py). The most common reason for withdrawing was moving (Supplementary Table 2). Remaining consistently engaged in care was not common (3 per 100 py).

One in 5 participants (n = 383) died during the study period. Death was twice as likely after transitioning through LTFU as those who remained engaged in care (5 compared to 2 events per 100 py) (Figure 1). Despite linkage with provincial registries and other attempts to determine the cause, 1 in 4 participants had unknown causes of death (Table 2). For those in care until death, overdose (18%), infections other than HIV (16%), and end-stage liver disease (ESLD) (12%) were the most common causes of death. Among people who were LTFU and then determined to have died, 36% had unknown causes of death, and the most commonly known cause were infections (17%), followed by overdose (12%) and ESLD (12%). Among the participants that transitioned from reengaged to death, unknown causes of death remained high (32%), followed by overdose (17%) and ESLD (12%).

**Table 2.** Causes of Death From Each Transition State

	Engaged to Death (Total = 233) n (%)	LTFU to Death (Total = 69) n (%)	Reengage to Death (Total = 81) n (%)
Overdose	42 (19)	8 (12)	14 (17)
Infections	37 (16)	12 (17)	4 (5)
ESLD (in. HCC)	29 (12)	8 (12)	10 (12)
Cancer	23 (10)	5 (7)	8 (10)
CVD	20 (9)	2 (3)	9 (11)
Trauma/suicide	13 (6)	1 (1)	4 (5)
Pulmonary	7 (3)	1 (1)	1 (1)
HIV/AIDS	7 (3)	5 (7)	0
Other	3 (1)	2 (3)	4 (5)
Unknown	49 (21)	25 (35)	26 (32)

Abbreviations: CVD, cardiovascular disease; ESLD, end-stage liver disease; HCC, hepatocellular carcinoma; LTFU, Lost to follow up.

### Transitioning to LTFU

Factors associated with increased risk of transition from engaged to first LTFU episode were having a detectable HCV RNA (adjusted hazard ratio [aHR], 1.37; 95% confidence interval [CI], 1.13–1.67), no previous history of HCV treatment (aHR, 1.29; 95% CI, 1.09–1.52), evidence of HCV treatment but no SVR results (aHR, 1.99; 95% CI, 1.56–2.53), and recent incarceration (aHR, 1.94; 95% CI, 1.58–2.40) (Table 3). Although older age (0.76; 95% CI, .71–.81) and significant liver fibrosis (0.76; 95% CI, .63–.91) decreased the risk of transitioning from engaged to LTFU. Similar trends persisted among people who were LTFU multiple times (transitioned from reengaged in care to LTFU), in addition to being Indigenous, which increased the risk of being LTFU after being reengaged (aHR, 1.88; 95% CI, 1.49–2.40) (Table 2).

**Table 3.** Multistate Models Evaluating Factors Associated With LTFU and Reengagement

	Engaged to LTFU	Reengaged to LTFU	LTFU to Reengaged
Age (per 10 y)	**0.76 (95% CI, .71–.81)**	**0.81 (95% CI, .72–.9)**	**0.86 (95% CI, .79–.94)**
Indigenous	**1.09 (95% CI, .91–1.29)**	**1.88 (95% CI, 1.49–2.4)**	**1.28 (95% CI, 1.04–1.59)**
Sex (ref: male)			
Female	0.99 (95% CI, .85–1.15)	0.97 (95% CI, .78–1.2)	0.87 (95% CI, .73–1.04)
Men who have sex with men (ref: Men)	1.14 (95% CI, .96–1.34)	1.11 (95% CI, .86–1.4)	1.01 (95% CI, .82–1.23)
Income (<$1500/m) (ref: ≥$1500/m)	1.02 (95% CI, .89–1.17)	0.97 (95% CI, .79–1.2)	**0.84 (95% CI, .72–.99)**
PWID	1.09 (95% CI, .95–1.26)	0.92 (95% CI, .74–1.10)	1.10 (95% CI, .94–1.30)
Incarceration	**1.94 (95% CI, 1.58–2.40)**	**1.45 (95% CI, .98–2.1)**	0.90 (95% CI, .69–1.19)
HCV RNA (ref: undetectable)			
HCV RNA (detectable)	**1.37 (95% CI, 1.13–1.67)**	0.99 (95% CI, .73–1.3)	1.19 (95% CI, .94–1.50)
HCV RNA (not done)	0.97 (95% CI, .80–1.16)	1.04 (95% CI, .80–1.4)	**1.45 (95% CI, 1.17–1.79)**
HIV viral load (<50 copies/mL) (ref: ≥ 50)	1.02 (95% CI, .88–1.17)	0.83 (95% CI, .66–1.0)	0.98 (95% CI, .83–1.17)
CD4 (per 100 cells/mm^3^)	0.99 (95% CI, .98–1.01)	**1.05 (95% CI, 1.02–1.1)**	**1.04 (95% CI, 1.01–1.06)**
Significant liver fibrosis (APRI >1.5)	**0.76 (95% CI, .63–0.91)**	1.24 (95% CI, .91–1.7)	0.88 (95% CI, .71–1.09)
STI	1.00 (95% CI, .76–1.32)	0.76 (95% CI, .45–1.3)	1.20 (95% CI, .85–1.68)
HCV Treatment (ref: SVR)			
Treated but unknown SVR result	**1.99 (95% CI, 1.58–2.40)**	**1.80 (95% CI ,1.23–2.6)**	1.03 (95% CI, .77–1.37)
No history of treatment	**1.29 (95% CI, 1.09–1.52)**	**1.38 (95% CI, 1.11–1.7)**	**0.63 (95% CI, .52–.77)**
Failed	0.83 (95% CI, .61–1.13)	0.92 (95% CI, .61–1.4)	**0.58 (95% CI, .41–.83)**

Bold results indicate clinical or statisitcal significant results. Abbreviations: APRI, AST to platelet ratio index; CI, confidence interval; HCV, hepatitis C virus; LTFU, lost to follow-up; SVR, sustained virologic response. Results are adjusted hazards ratio, (95% confidence interval (CI)).

### Transitioning to reengaged

Being Indigenous (aHR, 1.28; 95% CI, 1.04–1.59) and not having an HCV RNA test result (aHR, 1.45; 95% CI, 1.17–1.79) were positive indicators of reengagement. In contrast, participants that were older age (aHR, 0.86; 95% CI, .79–.94), had no history of HCV treatment (aHR, 0.63; 95% CI, .52–.77), or previously failed HCV treatments (aHR, 0.58; 95% CI, .41–.83) were less likely to reengage after being LTFU (Table 3).

### Transitioning to Death

Participants could transition to the death state from 3 states: engaged, LTFU, or reengaged (Table 4). Consistently across each transition trajectory, being Indigenous was a significant predictor of death (aHR, 1.93; 95% CI, 1.32–2.83; aHR, 2.61; 95% CI, 1.33–5.10; and aHR, 2.38; 95% CI, 1.23–4.60) for each state, respectively. Low income was also a risk factor for death from the engaged state (aHR, 1.41; 95% CI, 1.00–1.99) and reengaged states (aHR, 1.83; 95% CI, .98–3.43). Of note, advanced liver disease (aHR, 1.62; 95% CI, 1.17–2.24) and older age (aHR, 1.44; 95% CI, 1.22–1.71) were the only factors associated with death for people transitioning from the engaged state. Undetectable HIV viral load had a protective effect (aHR, 0.51; 95% CI, .29–.89), whereas in contrast, no history of HCV treatment (aHR, 1.94; 95% CI, .90–4.22) increased the risk of death among those transitioning from being LTFU. Among those reengaged, a history of HCV treatment but no SVR result was associated with an increased risk of death (aHR, 2.85; 95% CI, 1.10–7.30).

**Table 4.** Multistate Models Evaluating Factors Associated With Death

	Engaged to Death	LTFU to Death	Reengaged to Death
Age	**1.44 (95% CI, 1.22–1.71)**	0.95 (95% CI, .70–1.28)	1.12 (95% CI, .83–1.50)
Indigenous	**1.93 (95% CI, 1.32–2.83)**	**2.61 (95% CI, 1.33–5.10)**	**2.38 (95% CI, 1.23–4.60)**
Sex (ref: male)			
Female	0.86 (95% CI, .62–1.21)	1.20 (95% CI, .69–2.09)	0.56 (95% CI, .31–1.01)
Men who have sex with men (ref: men)	0.74 (95% CI, .50–1.08)	0.92 (95% CI, .39–2.19)	0.45 (95% CI, .20–1.03)
Income (<$1500/m) (ref: ≥$1500/m)	**1.41 (95% CI, 1.00–1.99)**	1.24 (95% CI, .65–2.37)	**1.93 (95% CI, .98–3.43)**
PWID	1.26 (95% CI, .92–1.74)	0.64 (95% CI, .36–1.14)	1.24 (95% CI, .75–2.03)
Incarceration	0.66 (95% CI, .35–1.24)	1.43 (95% CI, .53–3.86)	0.70 (95% CI, .26–1.92)
HCV RNA (ref: undetectable)			
HCV RNA (detectable)	1.03 (95% CI, .65–1.65)	1.37 (95% CI, .53–3.58)	1.35 (95% CI, .65–2.80)
HCV RNA (not done)	1.24 (95% CI, .80–1.92)	1.58 (95% CI, .63–3.99)	0.78 (95% CI, .39–1.59)
STI	1.31 (95% CI, .65–2.61)	0.28 (95% CI, .04–2.13)	0.40 (95% CI, .05–2.95)
HIV viral load (<50 copies/mL) (ref: ≥ 50)	1.06 (95% CI, .77–1.46)	**0.51 (95% CI, .29–.89)**	0.79 (95% CI, .46–1.37)
CD4 (per 100 cells/mm^3^)	**0.91 (95% CI, .86–.97)**	**0.94 (95% CI, .86–1.03)**	**0.87 (95% CI, .79–.96)**
Significant liver fibrosis	**1.62 (95% CI, 1.17–2.24)**	0.96 (95% CI, .48–1.94)	1.66 (95% CI, .92–3.00)
HCV treatment (ref: SVR)			
Treated but unknown SVR result	1.57 (95% CI, .80–3.10)	0.50 (95% CI, .06–4.06)	**2.85 (95% CI, 1.10–7.39)**
No history of treatment	1.21 (95% CI, .82–1.79)	**1.94 (95% CI, .90–4.22)**	1.29 (95% CI, .70–2.38)
Failed	0.64 (95% CI, .33–1.23)	2.16 (95% CI, .67–6.96)	0.94 (95% CI, .36–2.42)

Bold results indicate clinical or statisitcal significant results. Abbreviations: CI, confidence interval; HCV, hepatitis C virus; PWID, people who inject drugs; STI, sexually transmitted disease; SVR, sustained virologic response. Results are adjusted hazards ratio, (95% confidence interval (CI)).

## DISCUSSION

Despite universal healthcare access, we observed frequent and differential churn in and out of care among PWH and HCV. These findings highlight the common occurrence of LTFU and mortality in this population, revealing how traditional care cascades are limited and may not accurately reflect critical milestones. To address this issue, we used longitudinal methods to study patients’ trajectories and found several underlying factors associated with our transition states. Notably, recent incarceration and requiring HCV treatment emerged as significant factors. Even among individuals who initiated HCV treatment, a substantial number of participants disengaged from care after initiating HCV treatment but before their final SVR results were known. Moreover, people requiring HCV treatment were less likely to reengage in care. To meet World Health Organization elimination targets, interventions must be designed to reengage and treat patients for HCV. This longitudinal approach identified opportunities to intervene and reinforce sustained engagement in care, which was also found to have significant clinical implications because participants who experienced LTFU events were twice as likely to die compared to those who remained consistently engaged in care. Disturbingly, our findings exposed that regardless of the transition state, Indigenous people were more likely to die, which suggests the possible role of structural racism and the deeply rooted inequities faced by Indigenous communities in Canada even after engaging in clinical care.

LTFU affects each step of the care cascade, ultimately preventing patients from achieving and maintaining successful treatment outcomes and increasing the risks of morbidity and mortality [23]. This study is the first to employ multistate modeling to analyze engagement patterns among people with HIV and HCV, focusing on LTFU and mortality trajectories. Multistate models have been used to evaluate disease progression among people with HCV but not to examine care patterns. Several studies have explored the dynamic nature of HIV care using multistate models. For example, similar to the result of our study, Gillis and colleagues reported that PWID, younger age, females, and Indigenous people in Canada were more likely to transition to suboptimal care and had a decreased likelihood of transitioning back to optimal care [24]. Researchers from the Ontario HIV Trials Network took a nuanced approach using multistate models to evaluate various states of antiretroviral adherence. They found that stress and nonadherence were associated with transitions to less favorable states of care [25]. Using data from the Center for AIDS Research Network of Integrated Systems cohort, researchers used multistate models to evaluate the cyclical nature of (dis)engagement and reentry into HIV care of 31 009 patients enrolled in the United States from 1996 to 2014. They found that specific time points in the care continuum were associated with an elevated risk of transitioning out of care [10]. Together, these studies underline how multistate modeling can be used to evaluate the cyclical nature of individual patients compared to the traditional cascade of care.

Although the baseline median age of this cohort was only aged 45 years, 21% died during the follow-up period. We found differential death rates based on the care trajectory, but the known causes of death were relatively similar across the trajectories. Preventable deaths as a result of injection drug use (overdoses and infections) and ESLD (avoidable with timely HCV treatment) were the most common causes of death across all transition states. This finding is consistent with a recent study by Hamill and colleagues that evaluated mortality rates among 21 790 patients from 3 high-income countries successfully treated for HCV treatment and found drug- and liver-related causes were the main drivers of excess mortality [26]. The authors highlight the need for continued support and follow-up even after successful treatment to maximize the impact of HCV viral suppression. Similarly, our study highlights the importance of harm-reduction interventions in conjunction with optimal clinical care.

These findings also contribute to our previous body of evidence revealing health service and death inequities, particularly among incarcerated [27] and Indigenous populations [16, 28, 29]. The entrenched historical and ongoing discrimination against Indigenous peoples of Canada functions as a pervasive barrier to sustained engagement in the HIV and HCV care cascades [30, 31], ultimately leading to negative long-term health outcomes [32]. Driven by historical and ongoing systems of oppression and White supremacy, systemic discrimination against Indigenous peoples is unfortunately embedded in the Canadian healthcare system [33]. These barriers to care are pervasive within the Canadian healthcare system, including HIV and HCV care. A systematic review by Jongbloed and colleagues highlights the impact of historical, intergenerational, and lifetime trauma—including the lasting effects of residential schools, forced assimilation, and poverty—on engagement in care and, consequently, the achievement of viral suppression among Indigenous peoples with HIV in Canada, Australia, New Zealand, and the United States [30]. Our study found that Indigenous people were twice as likely to transition to death as non-Indigenous participants regardless of engagement patterns while controlling for other sociodemographic and clinical factors highlighting that structural racism persists.

Strengths of our study include a representative cohort of people living with HIV-HCV across Canada, set in a universal health care setting. The richness of the sociodemographic variables collected routinely by this cohort allows for the evaluation of ethnicity, income, injection drug use and incarceration, which is not readily available using administrative data. Although the cascade of care is a valuable framework for monitoring and improving healthcare outcomes, particularly in chronic diseases, it is important to acknowledge its limitations. Data availability and quality, lack of standardized definitions and metrics, and limited focus on upstream factors are among the critical challenges associated with the cascade of care. Upstream factors include housing, food insecurity, mental health, intensity of substance use, social support, and experiences of stigma and discrimination. Because these experiences are not discrete, an intersectionality approach may be needed to comprehensively evaluate the role of social determinants of health on the cascade of care. Contextual variations across populations and healthcare systems further complicate the applicability of the cascade. Our study uses a multistate framework to address many of the challenges of traditional cascades of care. Our model handled competing risks, incorporated sociodemographic covariates in addition to clinical factors, and integrated flexible calendar time to reflect changes in treatment and care over time.

Our study also has limitations. We used data from a prospective clinical cohort; although participants are recruited and expected to attend clinical visits concurrently, they may have attended clinic visits without a record of a cohort visit or vice versa. To mitigate the potential of missed clinical visits, we used a conservative definition of LTFU (>18 months between visits) and conducted a sensitivity analysis to evaluate varying definitions. On the other hand, participants of this cohort do receive a small incentive ($15–$20 CAD) to compensate for their time while completing a research visit. This incentive may have encouraged attendance to routine clinic visits, resulting in our LTFU rate being underestimated. Future studies will link the cohort participants to administrative databases to evaluate all encounters with the healthcare systems regardless of study participation. Our conservative definition of LTFU also accounts for interruptions in care during the beginning of the COVID-19 pandemic. As more follow-up data are collected, future studies will evaluate the specific effect of the pandemic on our outcomes of interest. Our model assumes that the probability of transitioning to a new state is not conditional on the history; we are currently exploring alternative methods for participants experiencing more than 2 LTFU events. Finally, our study focuses on a population of patients already engaged in clinical care; therefore, it is only generalizable to patients diagnosed and linked to clinical care.

In conclusion, this study provides valuable insights into the patterns of disengagement and mortality among individuals coinfected with HIV and HCV. The frequent transitions between different states of care engagement and the substantial mortality rate emphasize the need for targeted interventions that tackle disruptions in care. It is essential to acknowledge and actively work to dismantle the structural racism within our healthcare system and increase harm reduction to mitigate preventable deaths and ensure equitable outcomes for all populations.

## Supplementary Material

ofae239_Supplementary_Data

## Supplementary Data

Supplementary materials are available at *Open Forum Infectious Diseases* online. Consisting of data provided by the authors to benefit the reader, the posted materials are not copyedited and are the sole responsibility of the authors, so questions or comments should be addressed to the corresponding author.

## Notes

***Financial support*.** This work was supported by the Fonds de recherche du Québec - Santé (FRQS) Réseau sida/maladies infectieuses, the Canadian Institutes for Health Research (CIHR) [FDN-143270 to M. B. K.]; and the CIHR Canadian HIV Trials Network (CTN) [CTN222 to M. B. K]. A Queen’s University Research Initiation Grant supported the analysis [6037907 to S. S]. D. D. was supported by a summer student fellowship from the Canadian Network on Hepatitis C (CanHepC) and the CIHR; CanHepC is funded by a joint initiative of the CIHR [NPC-178912] and the Public Health Agency of Canada. E. M. is supported by a salary award from the Fonds de recherche du Québec-Santé (Chercheur de merite) and Canada Research Chairs (Tier 1). V. M. L. is supported by salary award from the Fonds de recherche du Québec-Santé (Junior-2). D. P. is supported by Canada Research Chairs (Tier 2). M. B. K. is supported by Canada Research Chairs (Tier 1). The funders had no role in the production of this manuscript. T. T., J. C., C. C., J. G., S. W., and A.W. do not have funding sources to disclose.
