# Supplementary material for: Frequent Disengagement and Subsequent Mortality Among People With HIV and Hepatitis C in Canada: A Prospective Cohort Study
Source: Open Forum Infect Dis. 2024 Apr 25;11(5):ofae239. doi: 10.1093/ofid/ofae239 (PMC11127478; doi:10.1093/ofid/ofae239)
Supplement: ofae239_Supplementary_Data [file ofae239_supplementary_data.docx]

**SUPPLEMENTAL**

Supplemental Table 1. Transition rates (/100 py) based on various LTFU defections at 9, 12, 18 and 24 month-intervals

|  | 9 months | 12 months | 18 months | 24 months |
| --- | --- | --- | --- | --- |
| Engaged to Lost | 40 | 24 | 13 | 9 |
| Reengaged to lost | 60 | 42 | 29 | 22 |
| Engaged to death | 2 | 2 | 2 | 3 |
| Lost to death | 4 | 5 | 5 | 5 |
| Reengaged to death | 3 | 4 | 5 | 7 |

Supplemental Table 2. Reasons for Withdrawal

|  | Engaged to withdrawn (n=48) | Lost to  withdrawn (n=60) | Reengage to withdrawn (n=31) |
| --- | --- | --- | --- |
| Unknown | 31% | 18% | 39% |
| Moving | 33% | 43% | 26% |
| No interest in the study | 35% | 28% | 16% |
| Sustained Virologic Response | 0% | 10% | 19% |

**Supplemental Figure 1. Calendar time (Spline)
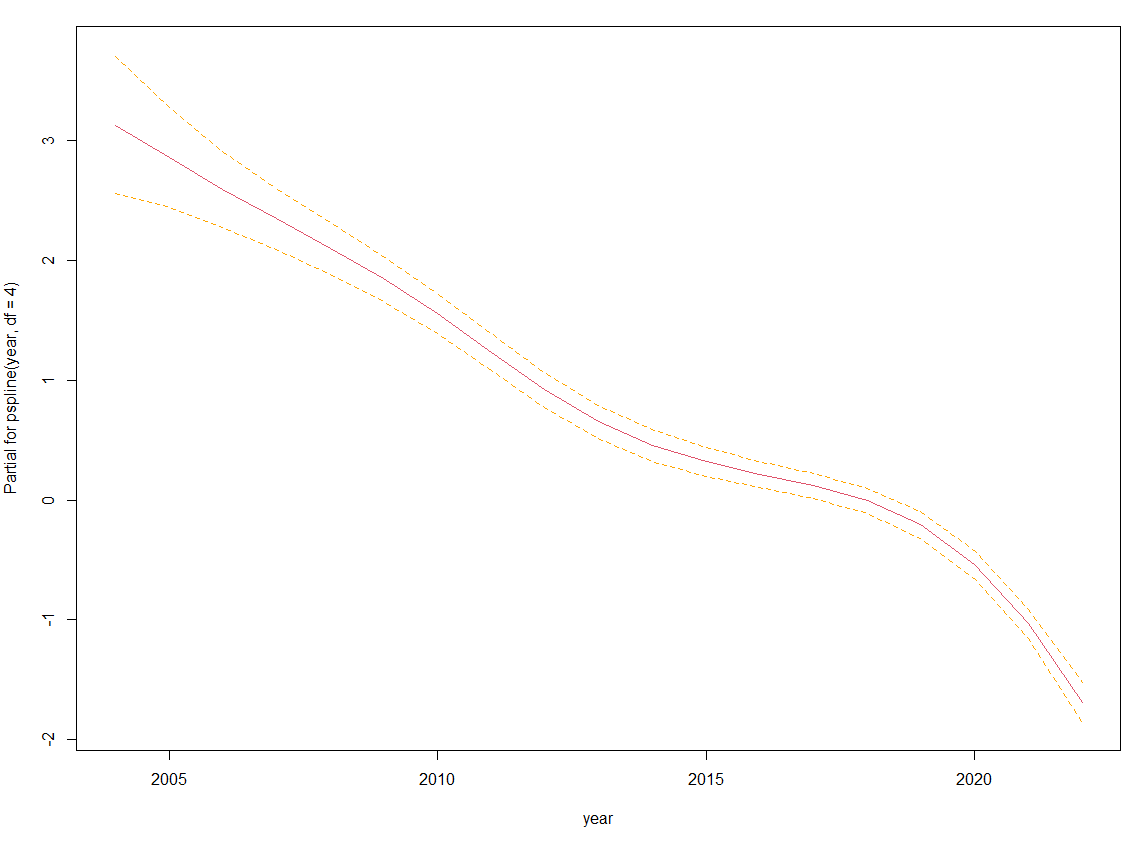
**
